# Peptidomic Analysis Reveals Temperature-Dependent Proteolysis in Rainbow Trout (*Oncorhynchus mykiss*) Meat During Sous-Vide Cooking

**DOI:** 10.3390/proteomes12040036

**Published:** 2024-11-27

**Authors:** Miyu Sakuyama, Yuri Kominami, Hideki Ushio

**Affiliations:** Department of Aquatic Bioscience, Graduate School of Agricultural and Life Sciences, The University of Tokyo, 1-1-1, Yayoi, Bunkyo-ku, Tokyo 113-865, Japanhushio@g.ecc.u-tokyo.ac.jp (H.U.)

**Keywords:** sous-vide cooking, proteolysis, peptidome, terminome

## Abstract

Sous vide, a cooking method that involves vacuum-sealed fish at low temperatures, yields a uniquely tender, easily flaked texture. Previous research on sous-vide tenderization has focused on thermal protein denaturation. On the other hand, the contribution of proteases, activated at low temperatures in fish meat, has been suggested. However, the details of protein degradation remain unclear. This study employed SDS-PAGE/immunoblot and peptidomic analysis of rainbow trout to assess proteolysis during sous-vide cooking. The results from SDS-PAGE and immunoblot analysis indicated reduced thermal aggregation of sarcoplasmic proteins and increased depolymerization of actin under low-temperature cooking conditions. A comparison of the peptidome showed that the proteolysis of myofibrillar proteins was accelerated during sous-vide cooking, with distinct proteases potentially activated at different cooking temperatures. Terminome analysis revealed the contribution of specific proteases at higher temperatures in rainbow trout. The results of this study demonstrate the thermal denaturation of sarcoplasmic proteins and proteolysis of myofibrillar proteins in rainbow trout meat during sous-vide cooking and its temperature dependence. The methodology in the present study could provide insights into the optimization of cooking conditions for different fish species, potentially leading to improved texture and quality of sous-vide products.

## 1. Introduction

The global population has been continuously increasing, and the global consumption of seafood is also increasing accordingly. Seafood has been shown to have excellent nutritional and functional properties due to omega-3 fatty acids, fish protein, and other components. Sous vide, a low-temperature cooking method, is gaining popularity alongside the expanding consumption of seafood, particularly with the rise in awareness of mi-cuit, a well-known sous-vide seafood dish. Sous vide involves cooking fish in a vacuum at low temperatures, resulting in a unique, tender texture that falls apart easily. Additionally, since the fish is heated in a vacuum, it retains flavors and nutrients that would otherwise be lost or diminished through conventional cooking methods [1]. Furthermore, the low-temperature cooking method helps maintain the vibrant color of the fish by preventing the denaturation of pigment proteins. Sous-vide cooking becomes easier with the increasing popularity of sous-vide cookers in households, making it a dish suitable for modern lifestyles.

The most distinctive feature of sous-vide cooking is its unique and soft texture. Numerous studies have shown that the thermal denaturation of actin, a protein that constitutes muscle fibers, is a key factor in the hardening of texture in various types of meat, including fish, beef, pork, and chicken [2,3]. It has been suggested that an increase in the hydrophobicity of protein due to thermal denaturation decreases the water-holding capacity of meat, resulting in hardening [4]. The denaturation temperature of actin in Atlantic salmon *Salmo salar* and rainbow trout (*Oncorhynchus mykiss*), both commonly used in sous-vide cooking, is approximately 78 °C [5,6]. It is also suggested that sous-vide cooking of fish meat at temperatures below the threshold suppresses actin denaturation, resulting in a softer texture [1].

Proteolysis also contributes to the tenderization of meat during sous-vide cooking [7,8]. The activation of cathepsin B and L in beef during sous-vide cooking has been demonstrated [7]. Many fish species have been reported to express proteases that are activated at 40–60 °C [9,10,11], suggesting that proteolysis may be involved in the softening of fish meat during sous-vide cooking. However, the progression of proteolysis in fish meat through sous-vide cooking has not been extensively investigated. The present study thus aimed to clarify the relationship between textural changes and proteolysis in fish meat during sous-vide cooking.

To explore the proteolysis in meat and fish meat during sous-vide cooking, peptidomic analysis can provide crucial information. It allows us to identify the substrate proteins and cleaved protein fragments [12]. This is a powerful method for investigating proteolysis in food processing, such as fermentation, aging, and *postmortem* changes [13,14,15,16]. Furthermore, the peptidome can reveal insights into the preferred subsites of endogenous proteases activated at the cooking temperature. The terminal sequences of the derived peptides exhibit distinct patterns corresponding to the cleavage site specificity of the proteases responsible for their generation [17,18]. Changes in the contributions of various proteases to the overall proteolysis can be discerned by comparing the comprehensive terminal sequences of cleaved peptides (terminomes) [19,20]. The present study performed peptidomic analysis to explore the proteolysis in rainbow trout meat during sous-vide cooking. Additionally, texture profile analysis (TPA) and SDS-PAGE analysis were conducted to assess the relationship between protein aggregation and proteolysis, as well as the resulting textural changes.

## 2. Material and Methods

### 2.1. Material

Fresh, never-frozen fillets of rainbow trout, sourced from seawater farms in Chile, were purchased at a local market (Toyosu in Tokyo, Japan). The skin and bones were removed from the fillets, and only the dorsal muscle was used for the experiments. This study did not involve any animal experiments or research on human subjects; therefore, ethical committee approval was not necessary.

### 2.2. Sous-Vide Cooking of Rainbow Trout Meat

Sous-vide cooking of the rainbow trout was performed according to a previous study with minor modifications [21]. The rainbow trout meat trimmed into WDH 60 × 50 × 25 mm was immersed in 10% NaCl brine solution at 5 °C for 1 h. After gently patting the surface of the rainbow trout meat to remove excess moisture, it was placed in a plastic bag and vacuum-sealed (VPF-S50, Iris Ohyama, Miyagi, Japan). The meat packed in the bag was equilibrated in ice water and then submerged in water baths (Thermminder Mini-80, TAITEC, Saitama, Japan) maintained at 52 °C, 65 °C, or 80 °C for 15 min each (referred to as SV-52, 65, or 80). Following heating, the rainbow trout meat was rapidly cooled in ice water and then brought back to room temperature. Both the heated and unheated (referred to as raw) rainbow trout meat samples were then subjected to TPA.

### 2.3. TPA

Cylindrical samples with a diameter of 20 mm were cored from the raw or cooked rainbow trout fillets and trimmed to a thickness of 10 mm by cutting from the side where the spine was located. Each sample was placed on the sample stage with the dark muscle facing down. The stress response of the meat during constant-speed compression was measured using a rheometer (FUDOH RHEOMETER NRM-2010-CW, Rheotech Co., Ltd., Tokyo, Japan) connected to a data logger (NR-HA08, KEYENCE, Osaka, Japan). An 80 mm diameter disc plunger was used to compress the samples to 65% of their original height at a speed of 1 mm/s, and the stress (gf) was recorded. Four TPA parameters were derived from the obtained TPA curves [22]. After TPA analysis, the samples were cut into small pieces, flash-frozen in liquid nitrogen, and stored at −80 °C until further processing.

### 2.4. SDS-PAGE and Immunoblot Analysis

A small portion of frozen tissue was placed in a 1.5 mL screw cap micro tube (Sarstedt K.K., Tokyo, Japan) containing 5 alumina HD beads (φ2 mm). An extraction buffer consisting of 20 mM KCl and 100 mM Tris-HCl (pH 7.4) with a protease inhibitor (cOmplete™ ULTRA Tablets, Mini, EASYpack Protease Inhibitor Cocktail, Merck, Darmstadt, Germany) was added at a tissue (mg) to buffer (µL) ratio of 1:9. The mixture was homogenized using a ShakeMan 6 homogenizer (Bio Medical Science Inc., Tokyo, Japan) (4200 rpm, 45 s with a 15 s interval, repeated twice) and then centrifuged (12,000 rpm, 4 °C, 60 min). The supernatant was collected and mixed with 4× Laemmli Sample Buffer containing dithiothreitol (Bio-Rad Laboratories, Inc., Hercules, CA, USA), according to Laemmli [23]. The sample was reduced by heating at 95 °C for 5 min and separated on a 2–15% gradient polyacrylamide gel (Multigel^®^II mini2/15, 17 well, Cosmo Bio Co., Ltd., Tokyo, Japan) at a constant 15 mA/gel. After electrophoresis, polyacrylamide gels were stained overnight with Bio-Safe™ CBB G-250 (Bio-Rad Laboratories, Inc.). For immunoblotting, proteins separated by SDS-PAGE were transferred onto polyvinylidene difluoride (PVDF) membranes (Immobilon-FL, Merck & Co., Rahway, NJ, USA). Membranes were blocked with 8% skim milk in phosphate-buffered saline containing 0.1% Tween 20 (PBST) for 1 h at room temperature. Following three washes with PBST, membranes were incubated overnight at 4 °C with anti-actin antibody AC-40 (ab11003, Abcam, Celbridge, UK). After washing with PBST, membranes were incubated for 1 h at room temperature with Alexa Fluor^®^ 680-conjugated anti-mouse IgG H&L antibody (ab175775, Abcam), followed by final washes with PBST.

Images of the SDS-PAGE band and immunoreactive proteins were acquired with the infrared imaging system (Odyssey Fc Imaging System, LI-COR, Lincoln, NE, USA) set for an excitation wavelength of 680 nm and a fluorescence wavelength of 700 nm. The molecular weight of each separated protein band was estimated by the relative migration distance.

### 2.5. Free Peptide Extraction

Approximately 100 mg of frozen sample was added to 1 mL of ice-cold 0.1% formic acid (FA)-2% acetonitrile (ACN) solution containing alumina HD beads (φ2 mm) to achieve a concentration of 50 mg/mL. The mixture was homogenized using a ShakeMan 6 homogenizer (4200 rpm, 45 s) and then centrifuged (12,000 rpm, 4 °C, 60 min). The supernatant (800 μL) was subjected to ultrafiltration with a 10 kDa-cutoff filter (Amicon^®^ Ultra 0.5 mL Centrifugal Filters (NMWL:10K), Merck & Co.) (12,000 rpm, 4 °C, 60 min). The resulting filtrate was collected, and 400 μL of the filtrate was desalted using SDB tips (GL-Tip SDB, GL Sciences, Tokyo, Japan) by following the standard protocol. The eluate was subjected to vacuum centrifugation before being used for LC-MS/MS analysis.

### 2.6. Peptidomic Analysis

Using the Ekspert™ 400 Autosampler 2, 1 µL of the sample was introduced and separated with the Ekspert™ nanoLC 415 system (AB SCIEX, Framingham, MS, USA). In the nano LC, 0.1% FA was used as Solvent A and 0.1% FA in ACN as Solvent B. The separation was performed on a ReproSil-pur column (75 µm × 50 cm C18-AQ, 3 µm, 120 Å, Dr. Maich GmbH, Ammerbuch, Germany) with an elution gradient of 200 nL/mL (from 98:2 to 90:10 Solvent A: Solvent B in 2 min, to 44:56 in 88 min, maintained for 10 min, and then to 20:80 in 2 min). The eluate from the column was introduced into the TripleTOF^®^ 5600 system (AB SCIEX) using the NanoSpray^®^ III (AB SCIEX). First, a shotgun analysis was performed using information-dependent acquisition (IDA). MS (400–1250 m/z) was full-scanned with an accumulation time of 250 ms, and for peaks exceeding a certain threshold, 20 MS/MS (100–1600 m/z) spectra per cycle were measured. The measurement time for each MS/MS was 250 ms, with one cycle taking 2.3 s. Next, quantitative analysis using SWATH^®^ acquisition was repeated three times. MS (100–1600 m/z) was full-scanned with an accumulation time of 0.05 s, and MS/MS (400–1250 m/z) spectra were measured in 34 steps with a Q1 transmission window of 25 Da. Each MS/MS was measured for 0.096 s, with one cycle taking about 3.4 s, and measurements were conducted from the start of the nanoLC gradient schedule to 100 min. The dynamic exclusion time was set to 12 s.

A database search of the results from shotgun analysis was conducted using ProteinPilot^®^ software 4.5 (AB SCIEX) with protein sequence information of Salmonidae (Taxonomy ID: 8015) obtained from NCBI (downloaded on 30 August 2022) as the reference. For the ProteinPilot^®^ results, PeakView^®^ software 2.2.0.11391 (AB SCIEX) was used for identification, quantification, and alignment of the mass spectra. Based on the identification results, an ion library corresponding to a 1% FDR was created for the proteins, and total ion chromatograms were extracted. Subsequently, SWATH^®^ analysis was performed on the MS data obtained from SWATH^®^ acquisition using the MS/MS ALL with SWATH^®^ Acquisition MicroApp in PeakView^®^ software. During the SWATH^®^ analysis, peptide information with confidence below 95% was excluded to obtain the quantification data for proteins and peptides.

### 2.7. Peptide Terminome Analysis

Sequence specificity in the peptide terminome was calculated from the peptides quantified in the SWATH analysis as described previously [13]. Specificity matrices representing the cleavage pattern of proteins were expressed as a heatmap using R v4.0.3 with the packages “seqinr” [24], “stringr” [25], “Biostrings” [26], and “ggplot2” [27] and the R Base Package [28].

### 2.8. Statistical Analysis

Dunnett’s test was conducted using the ‘DescTools’ package in R v3.4.37 to compare the TPA parameters of rainbow trout cooked by SV-52, 65, or 80 versus raw [29,30].

## 3. Results and Discussion

### 3.1. TPA

**Figure 1:**
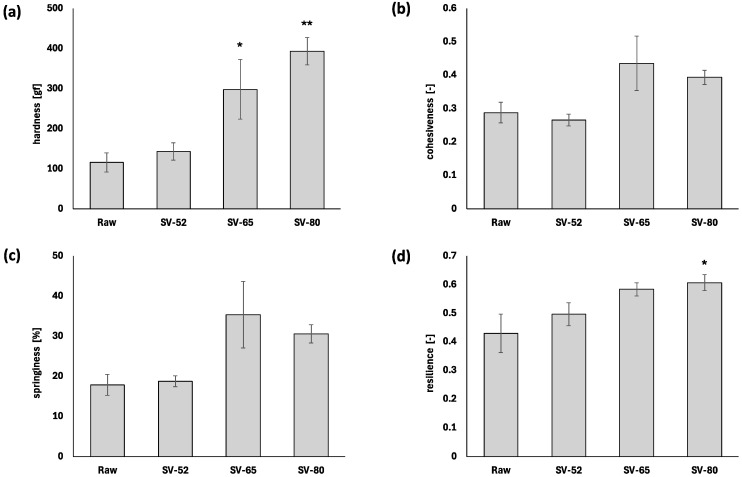
Comparison of TPA parameters in raw and sous-vide-cooked rainbow trout meat: hardness (**a**); cohesiveness (**b**); springiness (**c**); and resilience (**d**) (* *p* < 0.05, ** *p* < 0.01, Dunnett test vs. raw, *n* = 3).

The results of the texture profile analysis (TPA) are summarized in Figure 1. The hardness of the rainbow trout meat increased with increasing cooking temperature (Figure 1a). The rainbow trout meat sous vide at 80 °C (SV-80) exhibited significantly higher hardness compared to the raw meat (Dunnett, *p* < 0.01). Cohesiveness, springiness, and resilience also showed an increasing trend with higher cooking temperatures. A previous study showed a significant increase in the hardness of Atlantic salmon meat depending on cooking temperature by comparing raw meat and meat cooked at 29.4, 46.1, or 62.8 °C [6]. It also demonstrated significant effects of cooking on increasing the springiness of Atlantic salmon meat [6]. The differences in the effects of cooking on TPA observed in this study and the previous study can be attributed to the cooking method. We performed sous-vide cooking, while the previous study used a convection oven to cook salmon [6]. Sous-vide cooking can minimize water loss from fish meat, whereas cooking in a convection oven promotes water evaporation from the fish meat. Another previous study reported that there was no significant increase in the firmness of rainbow trout cooked in a sealed bag at temperatures ranging from 30 to 40 °C, while cooking at temperatures ranging from 60 to 70 °C increased firmness remarkably with rising temperature [5]. Combining these results indicates that the impact of cooking temperature on TPA in fish meat is related to dehydration during cooking. Therefore, the soft texture of fish meat after sous-vide cooking at moderate temperatures can be partially attributed to minimized water loss.

### 3.2. SDS-PAGE and Immunoblot Analysis

**Figure 2:**
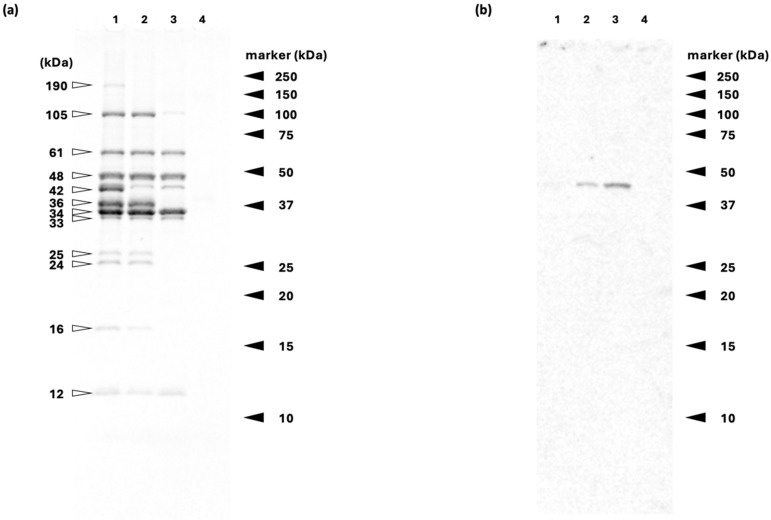
SDS-PAGE separation (**a**) and immunoreactive proteins (**b**). Separation of proteins extracted from raw and sous-vide-cooked rainbow trout meat with a low ionic strength buffer. Lane 1, raw; lane 2, SV-52; lane 3, SV-65; and lane 4, SV-80.

Figure 2a shows the SDS-PAGE separation of proteins extracted from rainbow trout meat using a low ionic strength buffer. The protein migration pattern varied according to cooking temperature. The separated protein bands, except for the ~190 kDa band, which is estimated to be myosin heavy chain, are mostly sarcoplasmic proteins [31]. According to a previous study [31], the ~24, 25, 33, 34, 36, 42, 48, 61, and 105 kDa bands contain triosephosphate isomerase, phosphoglycerate mutase, lactate dehydrogenase, glyceraldehyde phosphate dehydrogenase (GAPDH), aldolase, creatine kinase, enolase, phosphoglucomutase, pyruvate kinase, and glycogen phosphorylase, respectively. In addition to sarcoplasmic proteins, troponin T (~33 kDa) is also soluble in the low-ionic-strength buffer used in this study [32]. The decrease in band intensity is due to the thermal aggregation of the proteins during cooking at higher temperatures. In this study, a low-ionic-strength buffer was used to extract proteins from raw or cooked rainbow trout fillet, resulting in an extract primarily composed of sarcoplasmic proteins. The results indicated that most of the sarcoplasmic proteins thermally aggregate by cooking at 65 °C, and this aggregation is complete at 80 °C (Figure 2). These findings are consistent with a previous study using differential scanning calorimetry, which detected a thermal denaturation peak of sarcoplasmic proteins in rainbow trout meat cooked at lower temperatures (30–40 °C) but not at higher temperatures (60–70 °C) [5]. In other words, thermally aggregated sarcoplasmic proteins are not soluble under low-ionic-strength conditions; therefore, there were no significant bands in the extracts from SV-80.

Immunoreactive proteins with anti-actin antibodies in the proteins extracted from rainbow trout meat using the low-ionic-strength buffer are shown in Figure 2b. A single actin band was clearly observed in the SV-52 and SV-65 samples, while no band was observed in the raw and SV-80 samples. Actin solubilized in low ionic strength buffer, likely existing as G-actin, rapidly polymerizes into polar filaments upon the addition of KCl exceeding 100 mM [33]. The results of immunoblot analysis indicate that F-actin depolymerizes during cooking at 50–65 °C. The dissociation of F-actin by thermal denaturation has been suggested, but it remains unclear whether monomeric G-actin or short oligomers are released [33,34]. SDS-PAGE analysis of myofibrillar proteins extracted from sous-vide-cooked beef under high ionic strength conditions has revealed the presence of degraded protein fragments [7]. Another study using immunoblotting detected 26 kDa actin fragments in SDS-solubilized protein extracts from *postmortem* rainbow trout meat, which were not visible with CBB staining [35]. The rainbow trout used in this study was considered to be in a postmortem state of more than 5 days, as it was imported from Chile. However, actin fragments were not detected in our results (Figure 2b). This suggests that the amount of actin fragments may be too small to detect in the extracts under low ionic strength conditions. One possible explanation is faster breakdown into peptides or free amino acids or the binding of degraded actin to other proteins.

### 3.3. Peptidomic Analysis

**Figure 3:**
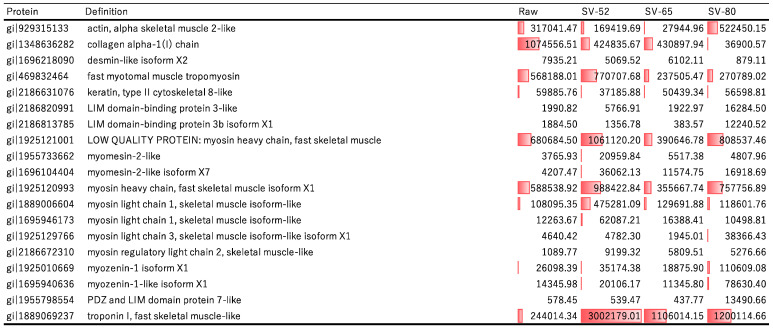
Quantification of the degraded muscle proteins related to meat texture in the rainbow trout meat. The data bar indicates the amount of each degraded protein.

The identification of degraded proteins and the quantification of peptides are summarized in Figure 3. A total of 595 free peptides were quantified (Figure S1) and identified as digests of 69 proteins. Many sarcoplasmic proteins, including pyruvate kinase, phosphoglycerate mutase, glyceraldehyde-3-phosphate dehydrogenase, creatine kinase, and aldolase, were identified as degraded proteins in the raw and SV-52-cooked rainbow trout meat. The degradation of the sarcoplasmic proteins was identified in fish meat, beef, and pork during *postmortem* [36,37,38]. Thus, the abundance of the peptides derived from sarcoplasmic proteins in raw rainbow trout meat is caused by *postmortem* proteolysis. The differences in the amount of these peptides between SV-52 and SV-65/80 indicate that they aggregate at higher temperatures but not aggregate at 52 °C.

The rainbow trout meat cooked by SV-52 was rich in fragmented peptides of myosin heavy chain and troponin I, while more actin fragments were found in SV-80 (Figure 3). The increase in the peptides derived from myosin heavy chain and troponin during SV-52 cooking indicates accelerated proteolysis. It has been suggested that the degradation of myosin heavy chain and troponin contributes to *postmortem* tenderization in beef and pork [39,40,41,42,43,44] and also in fish meat [41,42,43,44]. The proteolysis of myofibrillar proteins may contribute to the suppression of hardening in rainbow trout meat during SV-52 cooking. Previous research indicates that myosin unfolding at approximately 40 °C leads to a decrease in the elastic shear modulus (G′) in fish meat [6,45], while further heating induces gradually hydrophobic and disulfide bond formation, resulting in water loss and subsequent hardening [6,45]. Thermal denaturation of myosin at ~45 °C has also been demonstrated in rainbow trout meat [5]. Thus, myosin unfolding may also affect the texture of rainbow trout meat cooked by SV-52. Integrating the findings of the present study with previous research suggests that both the unfolding and proteolysis of myofibrillar proteins contribute to mitigating the hardening of rainbow trout meat sous vide at moderate temperatures [2].

The degradation of actin was accelerated during SV-80 (Figure 3). Thermal denaturation of actin above 65 °C in rainbow trout meat has already been shown [5]. It is important to note that this denaturation point was determined for F-actin, the filamentous form of actin, which is the predominant form in fish muscle. G-actin, the globular monomeric form of actin, is known to have a lower thermal denaturation point, typically 5–7 °C lower than that of F-actin [33]. A previous study showed that the proteolytic degradation rate of G-actin is higher than that of filamentous actin [46]. Additionally, as previously suggested [33,34], thermal denaturation may involve actin filament dissociation, potentially increasing actin susceptibility to proteolysis upon depolymerization. However, the degradation of actin during SV-80 cooking could not prevent the hardening. Thus, it is considered that the effects of thermal aggregation of actin were greater than degradation on the texture of the rainbow trout meat during SV-80 cooking. It has been suggested that troponin plays a role in stabilizing actin filaments together with tropomyosin [47]. The accelerated degradation of troponin in the SV-52 can promote dissociation of the actin filament. However, the results from immunoblot analysis indicated more monomeric actin in the SV-65 than in the SV-52. It is hypothesized that the thermal dissociation of actin, including its depolymerization into oligomers or monomers, is temperature-dependent. Our results highlight the complex interplay between thermal protein denaturation, proteolysis, and the resulting textural changes in fish meat during sous-vide cooking. Future studies should further investigate the interaction between thermal denaturation and thermal-activated proteolysis of myofibrillar proteins and their impact on textural changes in fish meat.

It has been reported that sarcoplasmic protease, which acts optimally at ~60 °C, degrades myofibrillar proteins in rainbow trout skeletal muscle [11]. In the present study, the core temperature of the rainbow trout meat may not have reached 65 °C and 80 °C in SV-65 and 80 treatments, respectively [48]. The proteolysis observed during SV-80 can, therefore, be attributed to the protease that acts optimally at ~60 °C [11]. Notably, the cleavage specificity of actin and troponin was similar between sous-vide cooking at 52 °C (SV-52) and 80 °C (SV-80), while that of myosin heavy chain and myozenin differed (Figure S1). This suggests that distinct proteases should be activated at these two different cooking temperatures.

**Figure 4:**
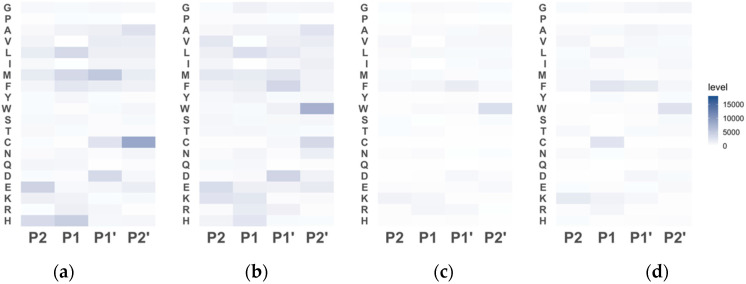
Sequence specificity in the terminome of the rainbow trout: raw (**a**); SV-52 (**b**); SV-65 (**c**); and SV-80 (**d**). Each column corresponds to the P2−P2′ subsite.

The results of the terminome analysis are shown in Figure 4. The sequence specificity in the terminome of raw and SV-52 was very similar. As discussed earlier, most peptides derived from sarcoplasmic proteins may not aggregate during SV-52 treatment. Thus, the sequence specificity in the terminome of raw and SV-52 is attributed to *postmortem* proteolysis. The intensity of Trp in P2′ subsite was higher in sous-vide-cooked rainbow trout meat than raw meat. Expression and high-activity cysteine proteases such as cathepsin B in skeletal muscle have been indicated in chum salmon *oncorhynchus keta* and Atlantic salmon, which are genetically close to rainbow trout [9,10]. The S2′ subsite of cathepsin B strongly prefers hydrophobic residues, and the binding pocket of cathepsin B tightly binds to Trp by π/π interactions [49]. A strong preference for Trp in the S2′ subsite has also been shown in µ-calpain, one of the cysteine proteases [49]. Cysteine proteases in rainbow trout meat could probably be activated during sous-vide cooking. The intensity of Cys in the P1 subsite was high only in SV-80. There has been no report on the expression of proteases that strongly prefer Cys in the S1′ subsite in rainbow trout skeletal muscle. However, our results indicate the expression of proteases that act optimally at higher temperatures and have specific cleavage preferences. The results from the peptidomic analysis elucidate that the proteolysis of myofibrillar proteins is induced during sous-vide cooking, but it is dependent on the cooking temperature. To determine the specific proteases that contribute to textural changes during sous-vide cooking of rainbow trout, further research is needed to identify and characterize the proteases expressed in rainbow trout meat, including their profiles and activity levels under different temperatures.

A previous study has suggested the potential of sous-vide cooking to enhance protein digestibility and generate bioactive peptides in pork [50]. This improvement in digestibility is attributed to both thermal unfolding and proteolysis of myofibrillar proteins. Moreover, variations in activated proteases during cooking can lead to taste differences by releasing diverse taste peptides or amino acids. While the relationship between thermal protein denaturation and sensory characteristics has been extensively studied, the connection between thermal proteolysis and sensory/nutritional characteristics remains poorly understood. The present study revealed accelerated proteolysis of myofibrils and the generation of peptides distinct from those produced by postmortem proteolysis, suggesting the activation of endogenous proteases during cooking. Further investigation is required to clear the impact of proteolysis in sous-vide cooking on both sensory and nutritional aspects.

Proteoform composition in fish skeletal muscle varies depending on individual physiological parameters such as growth and nutrition. Furthermore, triploid fish produced through aquaculture may exhibit greater proteome complexity compared to wild fish. These factors could influence proteolysis during sous-vide cooking of fish meat due to altered expression levels and activities of proteases prior to slaughter. In this study, the impact of proteoform alterations on proteolysis during cooking was not investigated. Proteomic/peptidomic studies could provide a valuable tool for exploring differences in proteolytic dynamics during cooking between wild and farmed fish in future research.

## 4. Conclusions

In this study, peptidomic analysis revealed that the proteolysis of myofibrillar proteins was accelerated in rainbow trout meat during sous-vide cooking. Notably, the proteolysis of myofibrillar proteins was accelerated during sous-vide cooking, with distinct proteases potentially activated at different cooking temperatures. Terminome analysis further supported this finding, indicating the temperature-dependent myofibrillar proteolysis in rainbow trout meat. Additionally, SDS-PAGE and immunoblot analysis of low ionic strength protein extracts indicated that F-actin partially dissociates during cooking at 52 or 60 °C.

Our results suggest that, in addition to minimizing water loss, inducing partial dissociation and proteolysis of myofibrillar proteins contributes to suppressing the hardening of rainbow trout meat during sous-vide cooking at lower temperatures. To our knowledge, this is the first study to report the changes in the peptidome of fish meat during sous-vide cooking. Further investigation of proteolysis during sous-vide cooking could help optimize cooking conditions for different fish species, potentially leading to improved texture and quality of sous-vide fish products.

## Data Availability

The datasets used and/or analyzed during the current study are available from the corresponding author on reasonable request.

## Supplementary Materials

The following supporting information can be downloaded at: https://www.mdpi.com/article/10.3390/proteomes12040036/s1, Figure S1. Quantification of the protein fragments in the rainbow trout meat. The data bar indicates the amount of each degraded protein. Uncharacterized or unnamed proteins were identified through BLAST search, and their identities were assigned as follows: *1 and 2, apolipoprotein A-II precursor; *3, fructose-bisphosphate aldolase A; *4, phosphoglycerate mutase 2; *5, phosphoglycerate kinase 1; *6, parvalbumin isoform; *7, fructose-bisphosphate aldolase A; *8, L-lactate dehydrogenase A chain; *9, elongation factor 1-alpha; *10, ATP synthase subunit e, mitochondrial; *11, Parkinson’s disease protein 7 homolog; *12, keratin, type I cytoskeletal 18; *13, histone-lysine N-methyltransferase Smyd1 isoform X1; *14, alpha-aminoadipic semialdehyde dehydrogenase; *15, adenylate kinase isoenzyme 1; *16, cytochrome c oxidase subunit 6B1 isoform X1.
