# Supplementary material for: Peptidomic Analysis Reveals Temperature-Dependent Proteolysis in Rainbow Trout (Oncorhynchus mykiss) Meat During Sous-Vide Cooking
Source: Proteomes. 2024 Nov 27;12(4):36. doi: 10.3390/proteomes12040036 (PMC11679362; doi:10.3390/proteomes12040036)
Supplement: Supplementary file 1 [file proteomes-12-00036-s001.zip › proteomes-3210552-supplementary.pdf]

Protein	Definition	Peptide	raw	x52C	x65C	x80C
gi|2186657385	40S ribosomal protein S29-like	ANDIGFVKLD	5683.233305	1954.481986	2186.165206	12459.33993
gi|929315133	actin, alpha skeletal muscle 2-like	AGDDAPRAVFPSIVGRPRHQGVMV	8049.517626	2288.738282	1645.40536	13499.13757
		APEEHPTLLTEA	1248.376272	1766.960403	1848.961733	28573.24623
		ASSSSLEKSYEL	7753.415416	5311.857281	808.3432717	10967.66466
		DNGSGLVKAGF	1171.093598	3741.063195	1360.249065	196932.4188
		DNGSGLVKAGFAG	9842.88487	10768.34273	826.7961768	1223.365591
		DNGSGLVKAGFAGDDAPR	19000.8796	20922.65099	1768.048539	1333.131299
		DNGSGLVKAGFAGDDAPRAVFPSIVGRPRHQG	26221.87604	21959.28553	766.0002037	1782.911154
		DN[Dea]GSGLVKAGF	1965.750046	2203.186078	820.5572829	163920.2127
		GMGQKDSYVGDEAQSKRGILTL	1240.262899	2408.409041	1044.313881	65172.56627
		LRVAPEEHPTL	7641.790024	17777.85629	2866.111277	718.2324987
		MGQKDSYVGDEAQSKRGILT	15639.44288	21339.30833	2915.326198	987.7987782
		SLYASGRTTGIVL	1209.959539	1370.074456	1049.675299	30351.5212
		VCDNGSGLVK	164813.9341	39767.00465	4785.754642	3415.638866
		VCDNGSGLVKAG	28695.49286	4763.73896	1118.14763	1285.267352
		[PGQ]-QAMWITKQEYDEAGPSIVHRK	22546.79303	13031.209	4321.267085	2287.037627
gi|2186824278	adenylate kinase 2, mitochondrial-like isoform X1	AAFSSATAAPCK	3254.859119	3457.759058	1588.999742	1072.35409
gi|1696087873	AMP deaminase 1 isoform X2	FAEKVFASETKGEGVR	6026.2306	6582.081757	2027.925735	2394.476443
		FAEKVFASETKGEGVRDE	8622.51446	8319.256758	1426.064757	1342.78367
		PKVNMPGQKTDEHML	2591.651738	2937.922915	1723.447442	1519.109231
gi|2186648171	apolipoprotein A-I-1	YETISQAMKA	12377.51579	22344.24206	19302.76802	1385.336841
gi|2174013542	arylsulfatase I	PSCQPLFMFLSLQAVHT	43871.7068	30650.08736	16512.025	1979.736566
gi|929249712	ATP synthase subunit epsilon, mitochondrial	VAYWRQAGLSY	2152.165846	7738.71477	410.6077024	912.0517672
gi|1925065342	beta-enolase isoform X1	MILPIGASNFH	1555.035176	29515.49761	7530.325109	742.4456809
		MILPIGASNFHEAMR	12161.5123	25589.6406	1535.448392	930.5291122
		PIGASNFH	3552.555175	25965.3405	2650.295025	1415.629028
		[1Ac]-SIIKIHAREILD	5530.547942	11810.9652	1549.780522	461.0505961
gi|1925149796	beta-enolase isoform X1	IEEELGNKAKFAGKDYRHPKIN	11143.94934	29933.3835	6147.385238	4942.416822
		MILPIGASNFH	1555.035176	29515.49761	7530.325109	742.4456809
		MILPIGASNFHEAMR	12161.5123	25589.6406	1535.448392	930.5291122
		MRIEEELGNKAKFAGKDYRHPKIN	5369.385876	15536.70031	23956.85529	9109.796989
		PIGASNFH	3552.555175	25965.3405	2650.295025	1415.629028
		RIEEELGNKAKFAGKDYRHPKIN	9593.999951	16788.62607	4600.28059	4666.388533
		[1Ac]-SITKIHAREIL	101123.4362	467573.239	93138.36605	13544.31667
		[1Ac]-SITKIHAREILD	57764.9305	151959.1171	30369.99874	3365.850362
		[1Ac]-SITKIHAREILDSRGN	4863.029214	9877.17138	1378.218235	2117.989599
		[1Ac]-SITKIHAREILDSRGNPT	20297.88874	33010.15024	2810.461702	3577.478719
		[1Ac]-SITKIHAREILDSRGNPTVE	19970.00829	39831.79163	550.8989806	749.0011006
gi|1348636282	collagen alpha-1(I) chain	FIAQPAQEKAPD	9418.382699	3948.166814	6254.769803	836.327706
		FIAQPAQEKAPDPFR	28056.05022	21304.35272	22044.5091	2552.110106
		FIAQPAQQKAPDPFRHF	44835.76843	33893.19329	31944.84109	1149.64876
		IAQPAQEKAPD	45752.17152	10761.50379	8209.674399	5271.037214
		IAQPAQEKAPDPFR	127905.9696	62516.03105	53770.79932	1233.031431
		IAQPAQEKAPDPFRH	12155.58674	11904.72657	16146.52356	1613.020731
		IAQPAQEKAPDPFRHF	154949.5047	25778.43555	21844.82674	3003.238373
		IAQPAQEKSPDPFR	6988.398119	9099.480574	12133.87828	1179.755026
		IAQPAQEK[Oxi]APDPFR	8077.030477	10112.59041	13817.82476	2309.714392
		IAQPAQQKAPDPFR	18866.53678	38804.31315	23462.61831	707.2661617
		IAQPAQQKAPDPFRH	8441.447286	15040.51459	9427.249711	1812.907607
		IAQPAQQKAPDPFRHF	166345.3922	52060.64058	78846.6687	2325.179287
		IAQPAQ[Dea]EKAPDPFR	87929.67471	43589.81433	43830.06041	3783.945921
		IAQPAQ[Dea]EKAPDPFRHF	50152.98788	2967.72747	6565.231459	1010.802469
		IAQ[Dea]PAQEKAPD	29627.32735	13029.43455	4895.425862	3162.564438
		IAQ[Dea]PAQEKAPDPFR	120009.0683	58239.9429	50438.88357	3811.822703
		IAQ[Dea]PAQEKAPDPFRHF	155045.2102	11784.80534	27264.15817	1138.200113
gi|1695880559	creatine kinase M-type-like isoform X1	EKGEAIDGMIPAQK	20252.60438	10133.04566	4638.7959	12041.24697
		EKKLEKGEAIDGMIPAQK	36495.87359	14278.56357	7553.780769	7975.890276
		GEAIDGMIPAQK	374220.8834	81108.47999	13782.17817	3091.667169
		GVDNPGHPFI	6160.686173	10699.12135	3450.114828	19172.29039
		GVDNPGHPFIM	8013.158835	13151.52692	5511.169338	2238.274672
		GVDNPGHPFIMT	6816.094946	1970.852077	1800.230219	2820.260487
		KGGDDLDPAYVLSSRVR	2557.381015	2740.971552	593.0708153	27399.16737
		KGGDDLDPAYVLSSRVRTGRSIKG	1471.147027	2747.295359	2058.39549	46339.41159
		LFDKPVSPLL	17845.93491	15163.89371	9559.005931	22516.03954
		MEKKLEKGEAIDGMIPAQK	44864.08468	37869.35468	12623.77388	7320.856499
		MVEMEKKEEKGEAIDGMIPAQK	79372.19373	25149.09308	5882.631051	1679.651497
		MVEMEKKLEKGEAIDGMIPAQK	241165.1091	52780.74341	21417.22822	11090.74927
		MVEMEKKLEKGEAIDGM[Oxi]IPAQK	25542.11951	4171.328606	910.4725379	2116.527039
		MVEMEKKLEKGEAID[Oxi]GMIPAQK	22736.97319	4723.654676	2840.037856	1398.162232
		MVVDGVKLM	10208.90285	5916.375083	3978.500312	816.4572807
		M[Oxi]VEMEKKLEKGEAIDGMIPAQK	29294.44338	19065.1999	6202.879542	1351.669739
		TQTGVDNPGHPF	2145.077868	3097.618725	3557.124621	48502.30652
		TQTGVDNPGHPFI	2206.150385	1398.560244	1309.512087	18137.04819
		VEMEKKLEKGEAIDGMIPAQK	445778.7571	132270.925	50091.07005	22206.82615
		VEMEKKLEKGEAIDGMIPAQ[Dea]K	305157.7212	99161.59694	38375.69442	23412.11214
		VEMEKKLEKGEAIDGM[Oxi]IPAQK	19552.81368	3519.582374	3018.931394	2053.431195
		VEM[Oxi]EKKLEKGEAIDGMIPAQK	17526.70124	3570.460874	6807.255377	3227.041346
gi|514052103	creatine kinase, muscle	DDVIQTGVDNPGHPF	1032.251267	4184.493497	3424.245333	8206.259858
		DVIQTGVDNPGHP	8610.823077	16506.19068	13884.87805	1719.101706
		DVIQTGVDNPGHPF	18466.5329	77838.39366	49574.70924	7298.628572
		DVIQTGVDNPGHPFI	13497.7711	24295.28655	9013.043722	1369.066149
		DVIQ[Dea]TGVDNPGHPF	19082.19121	68279.02561	44055.43708	954.8696095
		EKGEAIDGMIPAQK	20252.60438	10133.04566	4638.7959	12041.24697
		EKKLEKGEAIDGMIPAQK	36495.87359	14278.56357	7553.780769	7975.890276
		FGNTHNNFK	2727.432337	6767.201258	1971.896702	3806.8478
		GEAIDGMIPAQK	374220.8834	81108.47999	13782.17817	3091.667169
		GVDNPGHPFI	6160.686173	10699.12135	3450.114828	19172.29039
		GVDNPGHPFIM	8013.158835	13151.52692	5511.169338	2238.274672
		GVDNPGHPFIMT	6816.094946	1970.852077	1800.230219	2820.260487
		KGGDDLDPNYVLSSRVRTGRSIKG	1495.413601	1481.710647	980.4538385	6947.279961
		KVEEEYPDLTKH	1773.031033	6436.490989	5173.66828	17496.7816
		LDDVIQTGVDNPGHPF	14825.76986	21041.59573	1442.604979	1722.857452
		LFDKPVSPLL	17845.93491	15163.89371	9559.005931	22516.03954
		MEKKLEKGEAIDGMIPAQK	44864.08468	37869.35468	12623.77388	7320.856499
		MVEMEKKEEKGEAIDGMIPAQK	79372.19373	25149.09308	5882.631051	1679.651497
		MVEMEKKLEKGEAIDGMIPAQK	241165.1091	52780.74341	21417.22822	11090.74927
		MVEMEKKLEKGEAIDGM[Oxi]IPAQK	25542.11951	4171.328606	910.4725379	2116.527039
		MVEMEKKLEKGEAID[Oxi]GMIPAQK	22736.97319	4723.654676	2840.037856	1398.162232
		MVVDGVKLM	10208.90285	5916.375083	3978.500312	816.4572807
		M[Oxi]VEMEKKLEKGEAIDGMIPAQK	29294.44338	19065.1999	6202.879542	1351.669739
		PFGNTHNNFK	33143.76587	29463.86155	23089.78297	41584.73189

		PFGNTHNNFKLN	31548.2876	12594.4962	40579.53231	22777.4376
		PFGNTHNNFKLNF	46262.27881	15054.37929	18880.12267	85577.50762
		PFGNTHNNFKLNFK	60792.48774	45931.8644	18956.94786	769.5898757
		PFGNTHNNFKLN[Dea]F	24476.14251	6604.103738	8344.377294	41300.93721
		PFGNTHNN[Dea]F	1451.415915	4827.537964	18166.9252	76737.00937
		PFGNTHNN[Dea]FKLNF	14019.88907	2656.828993	9501.685038	6121.909153
		PFGNTHN[Dea]NFKL	5032.74703	2249.978448	2071.032738	9154.517077
		PFGN[Dea]THNNFKLNF	34022.77048	10283.67906	13964.76821	45734.64966
		VEMEKKLEKGEAIDGMIPAQK	445778.7571	132270.925	50091.07005	22206.82615
		VEMEKKLEKGEAIDGMIPAQ[Dea]K	305157.7212	99161.59694	38375.69442	23412.11214
		VEMEKKLEKGEAIDGM[Oxi]IPAQK	19552.81368	3519.582374	3018.931394	2053.431195
		VEM[Oxi]EKKLEKGEAIDGMIPAQK	17526.70124	3570.460874	6807.255377	3227.041346
		VIQTGVDNPGHP	1006.143162	9870.219242	5246.42437	2250.637656
		VIQTGVDNPGHPF	9633.31579	18520.37002	14628.70273	1832.052571
gi|2047164070	cytochrome c oxidase subunit 6C-1	[1Ac]-SLAKPAMRGLL	33816.7705	11397.98695	15529.08883	1012.011479
gi|1955784929	cytochrome c oxidase subunit 7A-related protein, mitochondrial-like	MALYIAAQPRK	9553.8764	11347.64853	7832.997728	1509.824406
gi|929300798	cytochrome c oxidase subunit 7C, mitochondrial	FGSGFAFPFVVVRHQLLKK	12437.14857	23873.03543	17940.66186	388.0357722
gi|1925009539	cytochrome c oxidase subunit 8B, mitochondrial	FVTILGPSGYVLAHLEDYKHHS	71500.51501	98982.31149	20344.426	1287.281075
		ILGPSGYVLAHLEDYKHHS	12374.64697	18228.64927	7854.721434	1339.805563
		LSHKPAKHHLSVGEQAIAM	19588.38162	36097.00883	1242.890656	1056.060413
		LSHKPAKHHLSVGEQAIAMTAF	1481.322188	2924.06667	17018.26857	1105.544691
		VTILGPSGYVLAHLEDYKHHS	45479.74254	88690.91383	40019.68097	2120.811137
gi|929078078	cytochrome c oxidase subunit NDUFA4-like	MLSTVSRQLKSHPALVPLF	44609.05109	53782.44442	26951.18746	1522.28544
gi|1696218090	desmin-like isoform X2	IKTIETRDGEVVSESTQHQQD	7935.209168	5069.519074	6102.1068	879.1121234
gi|469832464	fast myotomal muscle tropomyosin	[1Ac]-MDAIKKKMQ	3893.627081	8383.893344	1408.557506	958.5320715
		[1Ac]-MDAIKKKMQML	28016.93373	26464.11968	60975.50451	87538.58656
		[1Ac]-MDAIKKKMQMLK	71132.27188	282690.7184	52468.01558	106723.7399
		[1Ac]-MDAIKKKMQMLKLDK	15334.78721	33930.08315	4733.885817	1463.824693
		[1Ac]-MDAIKKKMQMLKLDKEN	21504.2453	24890.25689	16832.98218	6520.516146
		[1Ac]-MDAIKKKMQMLKLDKENA	3909.024178	5505.72273	3322.381362	1573.581794
		[1Ac]-MDAIKKKMQMLKLDKENALDR	171571.2086	145347.8881	20863.52694	11872.11589
		[1Ac]-MDAIKKKMQM[Oxi]LK	3654.989043	18324.74099	7720.47543	10633.04491
		[1Ac]-MDAIKKKMQM[Oxi]LKL	2185.755126	1156.314288	997.1936686	3987.049213
		[1Ac]-MDAIKKKMQM[Oxi]LKLDKENALDR	40974.97927	31795.2937	4536.77809	2392.005303
		[1Ac]-MDAIKKKMQ[Dea]M	2200.874849	3571.696966	7763.330715	4590.040113
		[1Ac]-MDAIKKKMQ[Dea]MLK	5342.366629	30241.82411	16405.57135	18306.07629
		[1Ac]-MDAIKKKMQ[Dea]MLKLDKEN	9988.54475	3732.308476	7624.315295	1980.453794
		[1Ac]-MDAIKKKMQ[Dea]MLKLDKENALDR	112235.1557	91975.44051	17244.93194	5526.799862
		[1Ac]-MDAIKKKM[Oxi]QMLKLDKEN	7088.605198	4968.969524	6407.480944	2913.836463
		[1Ac]-MDAIKKKM[Oxi]QMLKLDKENALDR	69154.63777	57728.41114	8200.534428	3808.818223
gi|2174003298	fetuin B	FAEDPLFK	1777.131204	5760.498763	15703.49731	2165.475875
gi|2186784485	fructose-1,6-bisphosphatase isozyme 2	SPDDVNEYLDFVKKHK	7647.130847	8981.109211	7934.21964	1459.765737
gi|2186813116	fructose-bisphosphate aldolase A-like	ADESTGSVAKRFQ	4924.654121	11511.48687	8372.491896	1787.078074
		AGKPGNGKAAQEEFIKR	8729.775495	14681.27872	10528.0273	7101.593911
		AWAGKPGNGKAAQEEFIK	3691.880306	9877.90476	5568.719374	5740.065476
		AWAGKPGNGKAAQEEFIKR	19686.93466	58963.04495	20783.41237	18284.2031
		FSYGRALQASALKA	3592.220598	12313.45603	1232.83015	1750.192905
		FTADERAGPCIGG	22830.75538	13659.63873	1005.155581	944.6213086
		GNGKAAQEEFIKR	35112.40981	64530.90219	37153.59654	23993.75627
		IKVDKGVVPLAG	7134.075421	8915.787526	507.0212537	781.3787898
		ITFSYGRALQ	10769.52784	15499.19664	1390.656425	1285.278012
		ITSTTPSRLAIME	2157.850012	6421.969932	425.0377628	628.274413
		KITSTTPSRLAIMEN	8606.857306	10440.20762	898.1240079	1305.331732
		NSLACQGKY	9372.90944	8366.392158	2796.741016	13475.9824
		NSLACQGKYVS	8216.194051	3637.387652	1384.963815	1259.657808
		PHAFPFLTPDQKKE	8285.419321	10991.18347	19935.8958	22196.82521
		SLACQGKYVS	17766.80918	4040.896368	1034.468119	3533.698466
		SLACQGKYVSSG	5534.536342	8214.869045	1066.336217	6421.42349
gi|89143257	glyceraldehyde-3-phosphate dehydrogenase	AADGPMKGILGY	22740.10895	13795.999	1118.941058	95170.60988
		AADGPM[Oxi]KGILGY	1552.577858	2244.887138	1132.573596	19667.97974
		ADGPMKGILGY	6325.478241	2319.374907	480.6657593	13232.50021
		AFRVPTPNVSVV	1348.131324	1013.790139	746.0585405	17166.52845
		AFRVPTPNVSVVDL	2053.27301	1391.397847	909.436343	24642.76141
		DLMAHMATKE	3327.945648	5378.992971	3459.376442	2992.754609
		EFGYSNRVIDLMAHMATKE	11494.80164	8838.781586	187.2955344	231.2942478
		FGYSNRVIDLMAHMAT	5735.682439	5998.360713	577.759144	484.1586173
		FGYSNRVIDLMAHMATK	3347.754647	14195.00158	2926.649413	406.6532417
		FGYSNRVIDLMAHMATKE	113113.4317	169611.9092	68129.5821	1187.163327
		FGYSN[Dea]RVIDL	28780.09813	10934.61258	18329.19909	638.9957633
		GDTHSSIFDAGAGIALNDHFVK	12297.67399	24143.20592	899.5916743	860.9744364
		GYSNRVIDLMAHMATK	6732.348565	17318.19326	4519.661772	994.4164661
		GYSNRVIDLMAHMATKE	34996.37846	62265.19792	42665.19099	853.5733559
		IVAINDPFID	4839.06697	28512.67222	5723.68629	813.8266545
		IVAINDPFIDL	1625.898747	8028.050893	2010.769327	745.827638
		NEFGYSNRVID	12128.83891	15254.15499	5316.271544	1415.726571
		NEFGYSNRVIDL	5495.263859	19629.13236	13895.39117	739.2388986
		NRVIDLMAHMATKE	34471.87117	67389.37	61392.24676	4081.937761
		PANIKWGDAGATY	9446.107087	5853.024943	1846.370721	1065.673371
		RAAFHSKKGVEIVAINDPFID	6570.672381	23798.07155	2531.191086	2686.079364
		RAAFHSKKGVEIVAINDPFIDL	10801.85967	21219.91861	2583.165967	794.3997619
		RVIDLMAHMAT	90400.61601	106666.5962	9739.258763	4122.127998
		RVIDLMAHMATK	40896.2732	99401.49966	16266.76248	2678.862648
		RVIDLMAHMATKE	313998.1238	388740.9826	290570.0554	4977.126107
		RVIDLMAHM[Oxi]ATKE	13667.00045	23577.3536	24558.939	2699.220585
		RVIDLM[Oxi]AHMATKE	8449.166021	15350.69429	13216.05633	1687.649967
		SKKGVEIVAINDPFID	6314.859633	24155.13264	4902.157854	610.45615
		TVRLEKPASYDAIKKVVKA	3307.036435	5059.152353	2452.554478	35065.33547
		VGKVIPELNGKITGM	713.382718	2357.709369	1419.254279	9930.368062
		VKLVTWYDNE	2277.573342	2367.756415	3185.021357	1148.040831
		VKLVTWYDNEFGYSNRVID	19528.07224	18377.69312	954.7622503	569.1614725
		VKVGVNGFGRIG	57417.81404	140799.816	83191.98968	4111.918758
		VKVGVNGFGRIGRLVT	236738.1726	957138.8283	497142.1768	24045.29364
		VKVGVNGFGRIGRLVTR	88512.82873	301535.0547	130299.7394	24858.30248
		VKVGVNGFGRIGRLVTRAAFH	47716.14661	122548.8275	37605.85244	3512.448602
		VTWYDNEFGYSN	446.0165605	6063.511174	2795.842852	490.5699936
		VTWYDNEFGYSNR	2270.499545	16313.1393	664.4627251	388.2171103
		VTWYDNEFGYSNRVI	4695.233862	18535.07594	3712.881319	345.8344267
		VTWYDNEFGYSNRVID	13660.47574	99215.22341	11626.68025	545.7270267
		WYDNEFGYSNRVID	1230.123945	12201.34298	1866.153629	613.1315298
		YDNEFGYSNRVID	2410.333064	6730.076256	5830.886806	485.0134539
		YSNRVIDLMAHMAT	2363.219908	9002.427771	552.6959519	550.0613315
		YSNRVIDLMAHMATK	10222.76816	13878.81545	4131.684267	2002.68164

		YSNRVIDLMAHMATKE	59846.9563	117521.5686	93837.68776	1629.037453
gi|1889002340	glycogen phosphorylase, muscle form	[1Ac]-SKPFSDHDKRKQISVRG	43600.19764	82671.39752	67159.96732	79172.74388
gi|1889020270	glycogen phosphorylase, muscle form-like	LEKIPAPDEQLK	22213.00713	5049.696111	2723.20627	7156.336021
		[1Ac]-SKPLSDHDRKKQISVRG	27111.51987	67885.68009	45309.30016	66593.65207
gi|929059178	heat shock protein beta-11	TSPGAATESTAVEAEPKKD	2025.870328	8343.483821	3161.202514	3137.762429
gi|2186631076	keratin, type II cytoskeletal 8-like	[1Ac]-SVRKTTSYTVKSSSSGAAP	42928.70207	22302.14222	39605.63381	46599.10406
		[1Ac]-SVRKTTSYTVKSSSSGAAPR	10792.16365	6731.109703	7604.530625	7845.073185
		[1Ac]-SVRKTTSYTVKSSSSGAAPRS	6164.898236	8152.626447	3229.172273	2154.636801
gi|1211233578	L-lactate dehydrogenase A chain	ADLVESILKN	1425.650954	1681.202655	968.98204	6571.048283
		GNSGLTDIIHM	28965.02713	29595.25014	1053.606832	15762.15124
		VVNGAYEVIKL	998.2360229	1233.383332	411.3431185	9596.220596
		[1Ac]-TTKEKLITHVL	17351.69346	12858.80495	14186.829	150229.1881
		[1Ac]-TTKEKLITHVLA	14687.82532	5137.69182	7371.317631	3856.107225
gi|2186820991	LIM domain-binding protein 3-like	SAQILNLPEKA	1990.824173	5766.905306	1922.971684	16284.49953
gi|2186813785	LIM domain-binding protein 3b isoform X1	SLNGSAPWGFRL	1884.501208	1356.776072	383.5719841	12240.51762
gi|1925121001	LOW QUALITY PROTEIN: myosin heavy chain, fast skeletal muscle	AALYPPPPPEDKAKKGGKKK	135179.1286	119013.7297	79043.42304	337434.0045
		AGTVDYNITGWLEKNKD	18677.2276	18590.73144	12195.32998	19947.32417
		AGTVDYNITGWLEKNKDP	12605.06059	8012.175101	3641.645522	16011.36613
		ALYPPPPPEDKAKKGGKKK	15971.73211	27366.59293	12856.63656	35924.94999
		ASVIPEGQFMDNKKASEKLLGSIDVNHEDYKFGHTK	38794.64482	29110.84651	1521.64575	1826.822411
		GGSMQTVSSQFRENLHK	14772.08093	10166.79041	5454.064496	18482.60161
		GSIDVNHEDYKFGHTK	36697.13061	74965.49985	68449.93946	75873.45485
		GSIDVN[Dea]HEDYKFGHTK	28853.71738	56814.88199	54725.67478	61950.14154
		LGSIDVNHEDYKFGHTK	26431.45516	35150.96641	8152.490443	22420.8542
		LYPPPPPEDKAKKGGKKK	70459.42632	203504.7345	46059.97015	117560.2036
		N[Dea]ASVIPEGQFMDNKKASEKL	4350.837499	8899.948851	5290.719523	9394.392413
		N[Dea]ASVIPEGQFMDNKKASEKLLG	4878.116822	12808.12066	5050.150836	3786.092973
		N[Dea]ASVIPEGQFMDNKKASEKLLGSIDVNHEDYKFGHTK	117296.791	75820.13733	3808.371297	3595.326773
		RGKQAFTQQVEELKRAVEE	3806.895439	6261.062318	2569.380174	1178.835167
		SQNTSLLNTKKKLETDL	2551.123167	8940.12067	509.8237467	2446.989571
		TERGRKVAETELVDASER	2040.182102	76062.86884	3274.832043	2988.066858
		TERGRKVAETELVDASERVG	2128.452692	64271.36389	7577.205065	2489.520147
		TERGRKVAETELVDASERVGLLH	809.6881865	29892.27161	927.9863188	838.5977093
		TVDYNITGWLEKNKD	24492.06625	28898.64987	7239.033419	10884.84224
		VIPEGQFMDNKKASEKLLGSIDVNHEDYKFGHTK	48202.08759	81287.77283	21133.5215	10657.3116
		YAGTVDYNITGWLEKNKD	31530.16581	21338.98786	1913.184353	3143.00376
		YAGTVDYNITGWLEKNKDP	6399.550634	12002.18549	375.7251383	2368.357982
		YGKSGVKIL	15279.15394	2352.00048	11466.34664	1593.249663
		YLKGLVTARADGK	6275.781926	4584.962476	586.135373	1652.991536
		YNITGWLEKNKDPLN	1884.302487	8157.388701	873.8378385	1380.933846
		YPPPPPEDKAKKGGKKK	10317.69597	36845.40575	25949.70596	42707.22921
gi|1888994035	LOW QUALITY PROTEIN: myosin-binding protein C, fast-type-like	LVRALKDKEDGDEK	10669.8533	4826.383746	2592.858564	2346.256851
gi|1955733662	myomesin-2-like	AEPAYTVPAFRQRSADEIEEYQR	1731.272792	7966.084815	2319.634521	816.1792469
		TVPAFRQRSADEIEEYQR	2034.660582	12993.75046	3197.744892	3991.776411
gi|1696104404	myomesin-2-like isoform X7	TVPAFRQRSADEIEEYQR	2034.660582	12993.75046	3197.744892	3991.776411
		VATKTQVENIEIKREVQE	2172.808799	23068.37988	8377.003453	12926.91226
gi|1925120993	myosin heavy chain, fast skeletal muscle isoform X1	AALYPPPPPEDKAKKGGKKK	135179.1286	119013.7297	79043.42304	337434.0045
		ALYPPPPPEDKAKKGGKKK	15971.73211	27366.59293	12856.63656	35924.94999
		ASVIPEGQFMDNKKASEKLLGSIDVNHEDYKFGHTK	38794.64482	29110.84651	1521.64575	1826.822411
		GGSMQTVSSQFRENLHK	14772.08093	10166.79041	5454.064496	18482.60161
		GSIDVNHEDYKFGHTK	36697.13061	74965.49985	68449.93946	75873.45485
		GSIDVN[Dea]HEDYKFGHTK	28853.71738	56814.88199	54725.67478	61950.14154
		LGSIDVNHEDYKFGHTK	26431.45516	35150.96641	8152.490443	22420.8542
		LYPPPPPEDKAKKGGKKK	70459.42632	203504.7345	46059.97015	117560.2036
		N[Dea]ASVIPEGQFMDNKKASEKL	4350.837499	8899.948851	5290.719523	9394.392413
		N[Dea]ASVIPEGQFMDNKKASEKLLG	4878.116822	12808.12066	5050.150836	3786.092973
		N[Dea]ASVIPEGQFMDNKKASEKLLGSIDVNHEDYKFGHTK	117296.791	75820.13733	3808.371297	3595.326773
		RGKQAFTQQVEELKRAVEE	3806.895439	6261.062318	2569.380174	1178.835167
		SQNTSLLNTKKKLETDL	2551.123167	8940.12067	509.8237467	2446.989571
		TERGRKVAETELVDASER	2040.182102	76062.86884	3274.832043	2988.066858
		TERGRKVAETELVDASERVG	2128.452692	64271.36389	7577.205065	2489.520147
		TERGRKVAETELVDASERVGLLH	809.6881865	29892.27161	927.9863188	838.5977093
		TVDYNIAGWLEKNKD	15644.34096	11651.57389	2410.716343	3277.914811
		VIPEGQFMDNKKASEKLLGSIDVNHEDYKFGHTK	48202.08759	81287.77283	21133.5215	10657.3116
		YAGTVDYNIAGWLEKNKD	3077.606176	15003.18747	315.3514088	1270.586012
		YLKGLVTARADGK	6275.781926	4584.962476	586.135373	1652.991536
		YPPPPPEDKAKKGGKKK	10317.69597	36845.40575	25949.70596	42707.22921
gi|1695946173	myosin light chain 1, skeletal muscle isoform-like	[1Ac]-APKKDAKAAPAKKAEPAKAAAPAPAPEPDVVAAPPA	12263.67428	62087.20916	16388.40762	10498.81244
gi|1889006604		APKKDLKAAPAKKAEPAKAAAPAPEPEVVAAPPA	19452.65939	96154.63786	23518.03413	20400.74922
		APKK[3Me]DAKAAPAKKAEPAKAAAPAPEPEVVAAPPA	26848.32191	131661.6371	29643.56155	29829.4977
		[1Ac]-APKKDAKAAPAKKAEPAKAAAPAPEPEVV	6719.460111	12401.24027	21597.81148	15667.20321
		[1Ac]-APKKDAKAAPAKKAEPAKAAAPAPEPEVVAAPPA	55074.91028	235063.5749	54932.47302	52704.31277
gi|1925129766	myosin light chain 3, skeletal muscle isoform-like isoform X1	ADFMPMMEKV	2355.850569	2274.28541	669.029732	19129.15478
		RVFDKEGNGTVSGAELRI	2284.571197	2508.016043	1275.985181	19237.27339
gi|2186672310	myosin regulatory light chain 2, skeletal muscle-like	YVITHGEEKEE	1089.77207	9199.321663	5809.50593	5276.660644
gi|1925010669	myozenin-1 isoform X1	AVVYQHEIGARPSFNRTPIG	4789.600928	9637.930011	3878.361306	2552.838095
		FQKLVPSLG	6136.698587	13055.39296	6400.517209	3883.350032
		NRSATPFGGFDKA	4303.738196	3514.691676	2941.913649	48024.21606
		NRSATPFGGFDKASQL	3905.542829	3536.081087	2003.36601	26722.82899
		PDTHEVAHDEPELAVVY	2469.347643	1403.593988	1429.30225	10600.33582
		[PGQ]-QHEIGARPSFNRTPIG	4493.465324	4026.686815	2222.438592	18825.51072
gi|1695940636	myozenin-1-like isoform X1	FQKLVPSLG	6136.698587	13055.39296	6400.517209	3883.350032
		NRSATPFGGFDKA	4303.738196	3514.691676	2941.913649	48024.21606
		NRSATPFGGFDKASQL	3905.542829	3536.081087	2003.36601	26722.82899
gi|1925068010	nascent polypeptide-associated complex subunit alpha, muscle-specific form isoform X1	APAPPKPAPIKPVVA	1726.33074	679.1836823	1187.059688	31975.85125
gi|2047171706	Parkinson disease protein 7 homolog	AEVAATVKAPLVLKD	22429.84629	17052.1123	5955.824528	736.0094106
		EVAATVKAPLVL	20418.12892	17944.24729	4985.473446	296.2735057
		EVAATVKAPLVLK	52850.63533	65408.43958	22946.95155	444.3104853
		EVAATVKAPLVLKD	291424.1756	228944.4151	221709.765	1335.824228
		MGAEVAATVKAPLVLK	11147.4188	14271.84432	2530.576458	208.4740607
		MGAEVAATVKAPLVLKD	55185.38602	36527.70492	16883.22764	1515.441902
		M[Oxi]GAEVAATVKAPLVLKD	10447.81798	14048.42607	10597.25955	1497.899402
		VAATVKAPLVLKD	8072.531648	4131.570883	4107.411784	1201.799033
gi|2186814327	parvalbumin beta 2	AADSFNHKAFFAKVGL	1253.768767	2186.729597	827.6397414	15059.86378
		AGKSNDDVKKAFY	2397.945887	12560.90864	4513.966726	13649.47558
		AGKSNDDVKKAFYV	908.3594083	1224.12554	1683.501644	7906.72785
		AGKSNDDVKKAFYVI	3980.413332	4036.162419	3299.105821	13046.24208
		KLFLQNFSASA	1996.386115	1996.554588	447.4641665	4829.565372
		KVGLAGKSNDDVKKAFYVI	1854.014317	1799.941727	1654.100517	5174.157574
		SASARALTDAETKA	3995.788122	4093.871974	7845.431836	30221.28552
gi|1955798554	PDZ and LIM domain protein 7-like	TLNGPAPWGFRL	578.4541604	539.4725729	437.7689599	13490.66202
gi|1696112025	phosphoglycerate kinase-like	GDVYVNDAFGTAHR	12602.80078	6150.508143	11145.24424	14294.09962

		KLSNKLTLDKVDVEGKRVI	546.8283692	2296.742986	9349.103363	21793.91858
		LGDVYVNDAFGTAHRAHS	17744.79932	6676.76447	1124.92956	6609.151726
		LLGKDVHFLKD	8300.71035	7138.804261	4385.57842	7018.336524
		[1Ac]-SLSNKLTLDKVD	8691.458992	4687.231766	3826.59859	7614.700072
		[1Ac]-SLSNKLTLDKVDVEGKR	71880.59061	56616.91119	61602.81421	62680.85503
gi|213515184	phosphoglycerate mutase 2-2 (muscle)	AETVKKAMEAVAAQGKAKK	9747.050219	6815.875905	1642.30555	2964.695504
		DAETVKKAMEAVAAQGKAKK	24133.93401	32773.64653	9335.833884	14462.3532
		DVNLKPVKPMAFLGDAETVKKAMEAVAAQGKAK	73529.72413	46232.9685	1494.802649	1310.542744
		DVNLKPVKPMAFLGDAETVKKAMEAVAAQGKAKK	792192.3588	460695.0064	34442.89112	2828.515605
		FLGDAETVKKAMEAVAAQGKA	10925.44904	7488.403325	5137.79139	5796.259702
		FLGDAETVKKAMEAVAAQGKAK	77155.00607	53340.24876	43109.27592	43946.81367
		FLGDAETVKKAMEAVAAQGKAKK	272418.8202	180604.4649	120744.6281	179326.512
		FLGDAETVKKAMEAVAAQ[Dea]GKAK	13436.45942	7716.642306	11245.13728	10409.13137
		FLGDAETVKKAMEAVAAQ[Dea]GKAKK	18818.16098	13653.12145	8246.44163	26662.27169
		GDAETVKKAMEAVAAQGKAK	12144.83963	10517.10349	14580.40854	7787.11275
		GDAETVKKAMEAVAAQGKAKK	50924.38803	45412.07045	44174.13244	46833.39645
		GDAETVKKAMEAVAAQ[Dea]GKAKK	10508.77057	19319.12955	15355.06549	21886.79462
		LGDAETVKKAMEAVAAQGKA	8601.652689	4249.178597	7869.538028	11274.33352
		LGDAETVKKAMEAVAAQGKAK	19539.05589	23764.5673	22933.53315	24278.08691
		LGDAETVKKAMEAVAAQGKAKK	66185.26471	53772.96614	50605.72065	74344.44846
		LGDAETVKKAMEAVAAQ[Dea]GKAKK	7592.002765	8925.378815	4594.395254	6754.680041
		LNLPTGIPIV	37591.19348	37535.00797	930.1989669	295.4025978
		LNLPTGIPIVY	220291.4998	338682.1282	4631.871819	402.0562583
		LNLPTGIPIVYE	114349.0387	227569.158	14257.81722	247.5825311
		LN[Dea]LPTGIPIVY	8623.305068	14178.14371	187.2121025	376.3409526
		[1Ac]-TTAHKLVIVR	2689.79676	10039.9659	2312.055361	1111.730025
		[1Ac]-TTAHKLVIVRHGESEWN	15914.525	21865.17303	2385.295182	1490.769263
		[1Ac]-TTAHKLVIVRHGESEWNQYN	15995.80349	17750.4384	4692.517606	6046.994623
		[1Ac]-TTAHKLVIVRHGESEWNQYNK	38088.95988	36557.28821	3988.111526	3462.453285
		[1Ac]-TTAHKLVIVRHGESEWNQYNKF	12256.85354	23127.12861	2152.03264	1589.123709
		[1Ac]-TTAHKLVIVRHGESEWNQYNKFCG	20158.23664	11155.50844	673.8105765	1122.148373
		[1Ac]-TTAHKLVIVRHGESEWNQ[Dea]YNKF	5671.357466	18686.6651	1549.552003	947.2692788
gi|1955729958	pollen-specific leucine-rich repeat extensin-like protein 1	RAPYPQPPMSTARAP	1443.386229	25871.42132	7382.868615	8910.267071
gi|2047195994	pyruvate kinase PKM	AAMADTFLEH	36699.65837	33826.33798	3544.902607	1776.003661
		AAMADTFLEHM	25099.67213	19215.93503	2809.392746	694.5864218
		DIDSEPAVAR	8762.277271	7485.300283	2174.80212	1801.570038
		DIDSEPAVARNTG	39433.32899	14680.59214	807.0516352	1154.726876
		DIDSEPAVARNTGIICT	14915.45411	3045.924815	1192.035198	1606.587202
		HAAMADTFLEH	7865.311714	8657.971838	1024.992273	1238.59678
		HAAMADTFLEHM	88217.26747	43207.2608	5664.250973	888.1776336
		HAAM[Oxi]ADTFLEHM	9248.508433	8136.30711	470.6844691	1270.503616
		KVKEVGADF	8779.154602	12743.86482	1901.858625	1773.708545
		LDIDSEPAVARNTG	41875.71735	22085.78914	946.5711211	808.2999486
		LDIDSEPAVARNTGIICT	19037.80637	3458.719698	2127.018936	1771.611386
		SAFIQTQQLHA	7757.361487	7476.956551	2008.299899	1361.946731
		SKGKDMGEAFIQTQQLHAAM[Oxi]ADTFLEH	121528.5969	45076.48474	512.2886217	360.2316782
		SKGKDMGEAFIQ[Dea]TQQLHAAM	119163.8417	81352.87214	665.3294847	638.8467552
		SKGK[AAS]DM[Oxi]GSAFIQTQQLHAAM	171440.3717	98624.15507	1150.612939	1001.129678
		SKGK[AAS]DM[Oxi]GSAFIQTQQ[Dea]LHAAM	106072.8839	62348.78093	2467.581212	718.5147485
		YTNTMRVVL	8568.424503	3492.739332	1166.856403	722.5725773
		[1Ac]-SKGKDMGSAF	7774.130048	6374.236783	4415.70886	2301.128554
		[1Ac]-SKGKDMGSAFIQ	101267.8093	57018.34271	1060.684271	632.2335559
		[1Ac]-SKGKDMGSAFIQT	6795.333762	6480.963043	750.8501017	1421.846875
		[1Ac]-SKGKDMGSAFIQTQQLH	37209.8464	28898.43734	11277.24382	2136.146221
		[1Ac]-SKGKDMGSAFIQTQQLHA	221728.0162	110851.6876	1743.28524	2759.298433
		[1Ac]-SKGKDMGSAFIQTQQLHAA	50994.23914	37857.39698	1373.298527	1026.636806
		[1Ac]-SKGKDMGSAFIQTQQLHAAM	163607.4977	93049.76339	1555.090074	1455.926906
		[1Ac]-SKGKDMGSAFIQTQQLHAAMA	82435.63204	36198.55459	4035.521829	946.2465612
		[1Ac]-SKGKDMGSAFIQTQQLHAAMAD	28155.81583	16848.87413	3713.260066	548.4467694
		[1Ac]-SKGKDMGSAFIQTQQLHAAMADT	30252.70995	15863.45087	1572.92265	483.1390194
		[1Ac]-SKGKDMGSAFIQTQQLHAAMADTFLEH	921566.1272	335238.9031	2376.281321	723.786896
		[1Ac]-SKGKDMGSAFIQTQQLHAAMADTFLEHM	316032.0968	41731.10022	276.3899721	287.3631184
		[1Ac]-SKGKDMGSAFIQTQQLHAAMADTFLEHM[Oxi]	52620.42331	11702.11312	624.2166461	332.0831834
		[1Ac]-SKGKDMGSAFIQTQQLHAAM[Oxi]	30054.46727	20169.10315	607.0054469	686.08386
		[1Ac]-SKGKDMGSAFIQTQQLHAAM[Oxi]ADTFLEH	119767.633	44468.55228	812.1169824	476.7591155
		[1Ac]-SKGKDMGSAFIQTQQLHAAM[Oxi]ADTFLEHM	46849.92949	9970.1483	536.4571975	527.2979397
		[1Ac]-SKGKDMGSAFIQTQQLH[Oxi]AAMADTFLEH	439128.9779	138009.884	468.9948366	567.5586563
		[1Ac]-SKGKDMGSAFIQTQQ[Dea]LHA	140556.0503	65597.40506	825.9164652	2483.241733
		[1Ac]-SKGKDMGSAFIQTQQ[Dea]LHAA	33731.6854	23452.63474	1637.001787	810.4846829
		[1Ac]-SKGKDMGSAFIQTQQ[Dea]LHAAMADTFLEH	557505.3178	253951.2438	1907.032519	280.0567526
		[1Ac]-SKGKDMGSAFIQTQQ[Dea]LHAAMADTFLEHM	230554.5985	26238.27776	445.2244688	212.5307567
		[1Ac]-SKGKDMGSAFIQTQ[Dea]QLH	26275.43433	20447.0313	7809.062156	2292.938542
		[1Ac]-SKGKDMGSAFIQTQ[Dea]QLHAAM	102264.6044	60245.37222	1798.299272	869.6301961
		[1Ac]-SKGKDMGSAFIQTQ[Dea]QLHAAMADTFLEH	712640.7991	259112.751	1463.451752	676.8273032
		[1Ac]-SKGKDMGSAFIQTQ[Dea]Q[Dea]LHAAM	59366.90744	36446.37259	781.4864361	578.3837506
		[1Ac]-SKGKDMGSAFIQ[Dea]TQQL	38524.62445	21156.85293	9340.093163	1783.881212
		[1Ac]-SKGKDMGSAFIQ[Dea]TQQLH	23228.79102	19100.62679	7871.544133	1684.496769
		[1Ac]-SKGKDMGSAFIQ[Dea]TQQLHA	176088.1403	74481.42075	761.4003276	1639.005744
		[1Ac]-SKGKDMGSAFIQ[Dea]TQQLHAA	41136.94384	28895.44633	873.144014	1396.096882
		[1Ac]-SKGKDMGSAFIQ[Dea]TQQLHAAMAD	29026.45785	5038.39745	2195.378784	662.8004481
		[1Ac]-SKGKDMGSAFIQ[Dea]TQQLHAAMADTFLEH	684985.9192	308079.895	2752.478058	771.0781239
		[1Ac]-SKGKDMGSAFIQ[Dea]TQQLHAAMADTFLEHM	303987.4097	29123.3199	436.6459619	244.108155
		[1Ac]-SKGKDMGSAFIQ[Dea]TQQLHAAM[Oxi]ADTFLEH	104199.7023	40514.67693	583.6386179	1453.727908
		[1Ac]-SKGKDMGSAFIQ[Dea]TQQ[Dea]LHA	121615.5704	64648.39829	745.8127724	2398.447535
		[1Ac]-SKGKDM[Oxi]GSAFIQTQQLH	3063.845975	4555.669105	2086.754675	1735.077855
		[1Ac]-SKGKDM[Oxi]GSAFIQTQQLHA	20306.35513	24561.55904	1123.503145	2083.314561
		[1Ac]-SKGKDM[Oxi]GSAFIQTQQLHAAMADTFLEH	245889.0853	142452.3197	1539.355812	526.2144204
		[1Ac]-SKGKDM[Oxi]GSAFIQTQQLHAAM[Oxi]ADTFLEH	76577.54327	36532.89735	2098.453885	2087.788594
		[1Ac]-SKGKDM[Oxi]GSAFIQTQQ[Dea]LHAAMADTFLEH	494575.9017	209222.9756	1884.087137	412.0666261
		[1Ac]-SKGKDM[Oxi]GSAFIQ[Dea]TQQLHAAMADTFLEH	702060.549	261386.6747	980.6220802	755.0383969
gi|2047237057	sarcoplasmic/endoplasmic reticulum calcium ATPase 1 isoform X2	AMTGDGVNDAPALKK	7977.730876	11986.47706	6273.870583	8098.125665
		EITAMTGDGVNDAPALKK	23993.06823	27472.84284	16401.8037	9198.707221
		FLQGQDEITAMTGDGVNDAPALKK	16116.15981	22692.02881	5932.031842	1439.726687
		GQDEITAMTGDGVNDAPALKK	6924.478494	6653.230691	4971.889521	3536.48027
		MTGDGVNDAPALKK	10438.84093	12950.5521	7315.192093	5948.327048
		M[Oxi]ERAHTKTPAE	15359.18364	23895.17267	11697.74841	13460.93956
		[1Ac]-MENAHTKTPA	4552.757286	9320.745612	2254.666905	3860.194828
gi|2156935689	triosephosphate isomerase B	GAALKPEFVDIINAK	2661.588391	13238.35142	7261.361677	1739.915703
		GAALKPEFVDIINAKQ	65878.52388	119148.8451	74232.03801	42733.88941
		GAALKPEFVDIINAKQ[Dea]	52953.24029	95829.1825	44886.38276	34952.5075
		VGGAALKPEFVDIINAKQ	14647.56503	22945.42998	10579.94616	7409.447427
		[1Ac]-SRKFFVGGNWK	9094.624113	26649.49445	4494.642004	1900.251651

gi|1889069237	troponin I, fast skeletal muscle-like	ANLKEVKKEVKEEE	2745.074873	9050.024429	3971.808813	2440.661324
		DWRKNVDEQAGMDGRK	11078.69651	109359.1264	40644.65473	37800.84678
		DWRKNVDEQAGMDGRKK	2973.915499	48428.93088	19371.30715	12396.73992
		DWRKNVDEQAGMDGRKKK	62246.59832	770756.4167	297158.1427	224350.1815
		DWRKNVDEQAGMDGRKKKFE	7915.520227	79921.31055	23221.18954	24452.55651
		DWRKNVDEQAGM[Oxi]DGRKKK	31494.14119	152345.7401	116989.1197	98499.11612
		DWRKNVDEQ[Dea]AGMDGRK	10025.20128	84308.27103	22477.97672	31727.78774
		DWRKNVDEQ[Dea]AGMDGRKKK	51288.28003	604673.0268	236359.1472	232716.5685
		DWRKN[Dea]VDEQAGMDGRK	10212.07073	57214.18966	26476.7509	26508.89529
		DWRKN[Dea]VDEQAGMDGRKKK	5205.819517	98120.90827	31049.69313	19796.72462
		EVGDWRKNVDEQAGMDGRKKK	14922.99827	353721.2586	91213.53838	166123.2077
		EVGDWRKNVDEQAGMDGRKKKFE	3377.778267	56138.28347	12559.78759	23088.82128
		EVGDWRKNVDEQAGM[Oxi]DGRKKK	2986.795457	15476.47821	6879.738613	12651.18834
		EVGDWRKNVDEQ[Dea]AGMDGRKKK	14544.18683	319328.1268	83300.32806	169684.6511
		EVGDWRKN[Dea]VDEQAGMDGRKKK	3787.47464	127579.3808	31135.28053	56946.26762
		VGDWRKNVDEQAGMDGRKKK	9209.791331	115757.5338	63205.68982	60930.44139
gi|1925015639	uncharacterized protein LOC110537948[*1]	LDKILPAEE	14293.76819	41136.04035	58265.45737	4078.725682
gi|1888975821	uncharacterized protein LOC118376304[*2]	LDKVLPAEE	18468.55875	41362.54406	46189.36684	3594.179471
gi|1820196122	unnamed protein product[*3]	VGVIKAVDKKAASTGKVTK	13438.024	18925.15061	14483.0859	28282.61312
		VVIGHVDSGKSTTTGHL	10284.34076	3808.358704	3292.797161	5344.373772
		[1Ac]-GKEKIHINIVVIGHVDSGKSTTTGHLIYK	26376.8939	13533.32946	702.1418586	1456.312278
		AETVKKAMEAVAAQGKAKK	9747.050219	6815.875905	1642.30555	2964.695504
		DAETVKKAMEAVAAQGKAKK	24133.93401	32773.64653	9335.833884	14462.3532
		DANLKPVKPMA	15442.16198	4274.942148	1323.050692	1193.094998
		DANLKPVKPMAFLGDAETVKEAMEAVAAQGKAKK	422835.4396	300373.157	14363.04385	515.3596663
		DANLKPVKPMAFLGDAETVKK	20409.21246	15442.86187	3677.981498	3758.075797
		DANLKPVKPMAFLGDAETVKKA	11686.581	7540.929426	1525.536528	2106.648855
		DANLKPVKPMAFLGDAETVKKAME	9235.324608	7268.470038	666.7790678	958.1097968
		DANLKPVKPMAFLGDAETVKKAMEA	74130.3356	68582.74043	2504.849554	1944.765069
		DANLKPVKPMAFLGDAETVKKAMEAVAAQGKA	52358.7379	22894.64801	1475.883461	1336.571387
		DANLKPVKPMAFLGDAETVKKAMEAVAAQGKAK	347476.2107	231761.0286	4237.55018	2963.356223
		DANLKPVKPMAFLGDAETVKKAMEAVAAQGKAKK	1493720.298	1024219.217	56903.08915	4028.557565
		DANLKPVKPMAFLGDAETVKKAMEAVAAQ[Dea]GKAK	84387.17803	72100.52648	1021.260975	869.4052633
		DANLKPVKPMAFLGDAETVKKAMEAVAAQ[Dea]GKAKK	793468.049	629678.7427	21527.48096	1917.339564
		DANLKPVKPMAFLGDAETVKKAM[Oxi]EAVAAQGKAKK	21467.25761	20953.9445	14212.33581	22386.83991
		DANLKPVKPMAFLGDAETVKKSMEAVAAQGKAKK	438165.6119	307308.0227	15170.13491	1692.698896
		DANLKPVKPMAFLGDAE[KXX]TVKK[Oxi]AMEAVAAQGKAKK	25569.15594	16122.6402	450.405795	735.7760586
		DANLKPVKPM[Oxi]AFLGDAETVKKAMEAVAAQGKAK	26634.59056	24835.12615	17357.88975	13526.23625
		DANLKPVKPM[Oxi]AFLGDAETVKKAMEAVAAQGKAKK	265912.8049	531260.1265	28134.82424	18678.89558
		DANLKPVKPM[Oxi]AFLGDAETVKKAMEAVAAQ[Dea]GKAKK	40046.34068	16079.08236	2403.535789	3865.995195
		DANLKPVKPM[Oxi]AFLGDAETVKKAMQAVAAQGKAK	50244.62751	27373.0874	17961.4432	19891.34591
		DANLKP[Oxi]VKPMAFLGDAETVKKAMEAVAAQGKAKK	242399.8803	187288.9615	10891.96274	15818.23834
		DAN[Dea]LKPVKPMAFLGDAETVKKAMEAVAAQGKAKK	895617.5982	609317.1938	33482.05035	1539.284225
		FLGDAETVKKAMEAVAAQGKA	10925.44904	7488.403325	5137.79139	5796.259702
		FLGDAETVKKAMEAVAAQGKAK	77155.00607	53340.24876	43109.27592	43946.81367
		FLGDAETVKKAMEAVAAQGKAKK	272418.8202	180604.4649	120744.6281	179326.512
		FLGDAETVKKAMEAVAAQ[Dea]GKAK	13436.45942	7716.642306	11245.13728	10409.13137
		FLGDAETVKKAMEAVAAQ[Dea]GKAKK	18818.16098	13653.12145	8246.44163	26662.27169
		GDAETVKKAMEAVAAQGKAK	12144.83963	10517.10349	14580.40854	7787.11275
		GDAETVKKAMEAVAAQGKAKK	50924.38803	45412.07045	44174.13244	46833.39645
		GDAETVKKAMEAVAAQ[Dea]GKAKK	10508.77057	19319.12955	15355.06549	21886.79462
		LDANLKPVKPAAFLGDAETVKKAMEAVAAQGKAKK	135614.7129	91257.22667	4946.76462	1035.726796
		LGDAETVKKAMEAVAAQGKA	8601.652689	4249.178597	7869.538028	11274.33352
		LGDAETVKKAMEAVAAQGKAK	19539.05589	23764.5673	22933.53315	24278.08691
		LGDAETVKKAMEAVAAQGKAKK	66185.26471	53772.96614	50605.72065	74344.44846
		LGDAETVKKAMEAVAAQ[Dea]GKAKK	7592.002765	8925.378815	4594.395254	6754.680041
		LNLPTGIPIV	37591.19348	37535.00797	930.1989669	295.4025978
		LNLPTGIPIVY	220291.4998	338682.1282	4631.871819	402.0562583
		LNLPTGIPIVYE	114349.0387	227569.158	14257.81722	247.5825311
		LN[Dea]LPTGIPIVY	8623.305068	14178.14371	187.2121025	376.3409526
		[1Ac]-TTAHKLVIVR	2689.79676	10039.9659	2312.055361	1111.730025
		[1Ac]-TTAHKLVIVRHGESEWN	15914.525	21865.17303	2385.295182	1490.769263
		[1Ac]-TTAHKLVIVRHGESEWNQYN	15995.80349	17750.4384	4692.517606	6046.994623
		[1Ac]-TTAHKLVIVRHGESEWNQYNK	38088.95988	36557.28821	3988.111526	3462.453285
		[1Ac]-TTAHKLVIVRHGESEWNQYNKF	12256.85354	23127.12861	2152.03264	1589.123709
		[1Ac]-TTAHKLVIVRHGESEWNQYNKFCG	20158.23664	11155.50844	673.8105765	1122.148373
		[1Ac]-TTAHKLVIVRHGESEWNQ[Dea]YNKF	5671.357466	18686.6651	1549.552003	947.2692788
gi|642086835	unnamed protein product[*4]	ADSFNFKTFF	619.462228	384.0113676	511.9834792	8439.776109
		ASKSADDVKKAF	6698.405166	6182.084039	7780.057078	9316.554936
		ASKSADDVKKAFKV	1487.067349	16447.77305	5340.461526	38293.85684
		ASKSADDVKKAFKVI	1623.418534	1168.594474	797.9011016	40418.50021
		GFASKSADDVKKAFKV	1783.07614	981.9571538	798.4996161	41064.71783
		HTIGFASKSADDVKKAFKV	382.8140373	1839.072446	439.0949779	31503.58521
		IDEFAVLVKQ	16230.06544	18206.58891	12171.60503	512.9996723
		KAADSFNFKTF	1275.846277	917.2619871	1225.920895	216560.4672
		KAADSFNFKTFF	891.4343918	756.8412585	973.5639591	112309.9562
		KAADSFNFKTFFH	7799.662334	8776.795775	966.0189175	90533.21552
		KAADSFNFKTFFHTI	3190.538594	2528.887969	782.2584641	238361.1888
		KTFFHTIGF	2188.875593	1325.862578	2029.541045	134372.5466
		PKARVLTDAETKA	5522.22026	4860.345408	6359.240425	28556.07886
		PKARVLTDAETKAFLKA	2598.519086	2421.387185	876.8594339	9387.218078
gi|642100795	unnamed protein product[*5]	[1Ac]-ADKIKDAKIIF	61000.75798	30160.42501	11719.63034	3030.215346
gi|642102139	unnamed protein product[*6]	ADLVESILKN	1425.650954	1681.202655	968.98204	6571.048283
		GNSGLTDVIHM[Oxi]	3597.494513	2157.141896	2560.874238	10852.54278
		SGLTDVIHM	2108.941218	3638.594684	1130.954366	118921.046
		VVDGAYEVIKL	1718.518695	882.8594023	1245.558442	21206.70689
		[1Ac]-TTKEKLITHVL	17351.69346	12858.80495	14186.829	150229.1881
		[1Ac]-TTKEKLITHVLA	14687.82532	5137.69182	7371.317631	3856.107225
gi|642105999	unnamed protein product[*7]	[PGQ]-QTLVDGKVVSSSTETKERKQ	2817.08474	12070.79166	6508.470455	8395.003849
gi|642110900	unnamed protein product[*8]	ADESTGSVAKRFQ	4924.654121	11511.48687	8372.491896	1787.078074
		ANGKAAQEEFIK	18609.51946	5035.992946	8770.436085	16484.05413
		ANGKAAQEEFIKR	34828.70756	64153.93765	36546.31315	23354.0929
		ANGKAAQ[Dea]EEFIKR	25668.64446	49808.83772	26976.27012	20576.70489
		AWGGKPANGKAAQEEFIK	4492.778487	10433.53584	7819.433461	4651.069854
		AWGGKPANGKAAQEEFIKR	16722.02045	59093.7985	24047.11405	18292.17002
		FSYGRALQASALKA	3592.220598	12313.45603	1232.83015	1750.192905
		FTADDRAGPCIGG	42868.72428	22681.7117	1678.473157	1924.232737
		FTADDRAGPCIGGVIF	11554.53571	14221.30403	478.6659538	272.229441
		GGKPANGKAAQEEFIK	10398.37516	34674.45055	23086.19925	16139.52198
		GGKPANGKAAQEEFIKR	12039.54274	25853.77474	14182.19563	10332.35149
		GKPANGKAAQEEFIKR	26137.64334	45553.26017	33813.93974	30227.7402
		GKYVASGDSTAAGESL	1988.991992	7830.937011	3915.418159	2651.62314

		HETLYQKTDAGKTFPEHVKSRGWVVG	5726.911992	18117.78631	1085.497341	1858.844873
		IKVDKGVVPLAG	7134.075421	8915.787526	507.0212537	781.3787898
		ITSTTPSRLAIME	2157.850012	6421.969932	425.0377628	628.274413
		KITSTTPSRLAIMEN	8606.857306	10440.20762	898.1240079	1305.331732
		LTFSYGRALQ	10769.52784	15499.19664	1390.656425	1285.278012
		NSLACQGKY	9372.90944	8366.392158	2796.741016	13475.9824
		NSLACQGKYVA	9526.928634	3045.772375	1371.939512	2590.901278
		NSLACQGKYVASG	4751.471075	3589.733785	1371.120666	1164.545305
		PANGKAAQEEFIKRAL	1694.436605	16969.08109	1839.695046	2435.016338
		PHSFPFLTPDQKKE	13080.31052	14857.42892	15645.35125	16063.06772
		PHSFPFLTPDQKKEL	7761.182601	12254.00217	1654.512729	8949.083589
		PHSFPFLTPDQKKELS	37222.33432	59272.67348	10030.0067	2236.67731
		PHSFPFLTPDQKKELSD	19177.55662	18428.64637	4192.462974	4289.733846
		PHSFPFLTPDQKKELSDIALK	66807.29661	104769.7612	24980.47128	9046.701228
		SLACQGKYVASG	13953.95729	7092.617262	1018.559268	929.7251457
		VASGDSTAAGESL	3881.848776	4685.736578	1242.901772	2115.637168
		WGGKPANGKAAQEEFIK	2326.38409	4155.430774	1702.424996	2228.20774
		WGGKPANGKAAQEEFIKR	3001.543586	5835.983364	3492.123756	1467.29062
		WGGKPANGKAAQEEFIKRA	2999.689142	27911.29317	5517.275846	3708.204769
		WGGKPANGKAAQEEFIKRALA	2950.888977	8470.166173	7069.828773	1022.876076
		WGGKPANGKAAQEEFIKRALAN	2181.583336	18241.77912	1279.879566	1263.230263
		WGGKPANGKAAQEEFIKRALANS	1825.197056	23421.0336	2423.854312	1129.637542
		WGGKPANGKAAQEEFIKRALANSL	1103.825945	30025.14516	7336.1662	9111.104309
		WGGKPANGKAAQEEFIKRALANSLA	2295.759474	1482.58476	16974.29101	3824.31053
gi|642114152	unnamed protein product[*9]	NKPMAEASSVEDNIK	4090.545911	19348.85332	4327.049363	6234.574794
gi|642120901	unnamed protein product[*10]	FNVPMKDHNITNNQRIK	4205.536798	5934.144075	3585.268087	1786.58057
		GDVYVNDAFGTAHR	12602.80078	6150.508143	11145.24424	14294.09962
		GGGASLELLEGKVLPGVNA	10554.20747	17398.51659	5104.170461	5026.597868
		HLGRPDGNPMPDKFSLKP	10467.7483	3159.390059	1200.083586	2956.661735
		LEGKVLPGVNA	101063.9508	93233.92748	65207.69044	17827.1271
		LGDVYVNDAFGTAHRAHS	17744.79932	6676.76447	1124.92956	6609.151726
		LLEGKVLPGVNA	9719.008923	10178.8856	11278.29756	2389.732348
		LLGKDVHFLKD	8300.71035	7138.804261	4385.57842	7018.336524
		LSNKLTLDKVDVKGKR	3061.558024	3283.999583	2095.94476	4409.894882
		MSHLGRPDGNPMPDKFSLKPVAAELKS	84454.05523	25791.76925	13247.29921	33141.87837
		[1Ac]-SLSNKLTLDKVD	8691.458992	4687.231766	3826.59859	7614.700072
		[1Ac]-SLSNKLTLDKVDVK	8250.044254	8943.008387	7726.971705	8504.366016
		[1Ac]-SLSNKLTLDKVDVKG	93288.22668	62003.47238	37217.79452	80938.00855
		[1Ac]-SLSNKLTLDKVDVKGK	4582.894948	9183.209079	6998.791343	9823.798434
		[1Ac]-SLSNKLTLDKVDVKGKR	274946.7248	298209.2007	238227.7447	350315.0255
		[1Ac]-SLSNKLTLDKVDVKGKRV	132383.458	134488.6579	105970.6776	177398.0737
		[1Ac]-SLSNKLTLDKVDVKGKRVI	2514.387238	2381.065269	15668.57023	22584.95759
		[1Ac]-SLSNKLTLDKVDVKGKRVIM	25766.62197	12289.25106	1999.336401	12009.99792
		[1Ac]-SLSN[Dea]KLTLDKVDVKGKR	205497.2391	225889.023	174702.9792	259401.5714
		[CRM]-SLSNKLTLDKVDVKGKR	185204.1424	201744.423	156574.4904	233262.4435
gi|642124057	unnamed protein product[*11]	SKDLPLAQGIKFE	2946.588779	1805.085758	419.233649	16842.67412
gi|642127258	unnamed protein product[*12]	VPPVAVSPVIKTARW	8087.96442	17225.67479	3131.778004	923.4155994
		VPPVAVSPVIKTARWS	16270.85007	28667.41238	3173.465316	8816.526913
		VPPVAVSPVIKTARWSF	9452.380126	19323.6027	606.1277846	670.3304581
gi|642129305	unnamed protein product[*13]	ADESTGSVAKRFQ	4924.654121	11511.48687	8372.491896	1787.078074
		AWGGKPGNGKAAQEEFIKR	7286.171676	20659.01223	15244.88607	5185.33045
		AWGGKPGNGKAAQEEFIKRA	1823.429426	30451.02836	8069.631634	3533.920381
		FSYGRALQASALKA	3592.220598	12313.45603	1232.83015	1750.192905
		FTADERAGPCIGG	22830.75538	13659.63873	1005.155581	944.6213086
		GGKPGNGKAAQEEFIKR	23344.25713	37404.94956	29394.25212	26096.62976
		GNGKAAQEEFIKR	35112.40981	64530.90219	37153.59654	23993.75627
		HETLYQKTDAGKTFPEHVKSRGWVVG	5726.911992	18117.78631	1085.497341	1858.844873
		IKVDKGVVPLAG	7134.075421	8915.787526	507.0212537	781.3787898
		ITFSYGRALQ	10769.52784	15499.19664	1390.656425	1285.278012
		ITSTTPSRLAIME	2157.850012	6421.969932	425.0377628	628.274413
		KITSTTPSRLAIMEN	8606.857306	10440.20762	898.1240079	1305.331732
		NSLACQGKY	9372.90944	8366.392158	2796.741016	13475.9824
		NSLACQGKYVS	8216.194051	3637.387652	1384.963815	1259.657808
		PHAFPFLTPDQKKE	8285.419321	10991.18347	19935.8958	22196.82521
		SLACQGKYVS	17766.80918	4040.896368	1034.468119	3533.698466
		SLACQGKYVSSG	5534.536342	8214.869045	1066.336217	6421.42349
		WGGKPGNGKAAQEEFIKR	30865.44741	69207.91403	38891.69	26134.85542
		WGGKPGNGKAAQEEFIKRAL	1245.924114	21938.59125	1242.835024	829.1428036
		WGGKPGNGKAAQEEFIKRALAN	2519.201541	11971.98822	702.2194294	3404.802043
		WGGKPGNGKAAQ[Dea]EEFIKR	23192.00972	69202.70875	26158.40953	20727.23043
gi|642132354	unnamed protein product[*14]	EVAATVKAPLVL	20418.12892	17944.24729	4985.473446	296.2735057
		EVAATVKAPLVLK	52850.63533	65408.43958	22946.95155	444.3104853
		EVAATVKAPLVLKD	291424.1756	228944.4151	221709.765	1335.824228
		MGPEVAATVKAPLVLKD	26292.63161	28719.3974	22934.39094	552.9640262
		VAATVKAPLVLKD	8072.531648	4131.570883	4107.411784	1201.799033
gi|1820185295	unnamed protein product, partial[*15]	DDQIEAGSFPGKI	860.3968942	1174.703683	560.3878995	8056.696872
		VAKWDDQIEAGSFPGKI	4304.212125	5874.719557	64180.95169	11842.74948
		[1Ac]-SDVIEEKIKNY	1124.790676	1079.68839	1277.916349	51029.32623
gi|2493445	Major allergen Sal s 1	ASKSADDVKKAF	6698.405166	6182.084039	7780.057078	9316.554936
		ASKSADDVKKAFKV	1487.067349	16447.77305	5340.461526	38293.85684
		ASKSADDVKKAFKVI	1623.418534	1168.594474	797.9011016	40418.50021
		GFASKSADDVKKAFKV	1783.07614	981.9571538	798.4996161	41064.71783
		HTIGFASKSADDVKKAFKV	382.8140373	1839.072446	439.0949779	31503.58521
		IDEFAVLVKQ	16230.06544	18206.58891	12171.60503	512.9996723
		KEADIKTALEAC[2Ox]KAADTFS	4438.102097	1635.766333	3949.186106	22073.28325
		KTFFHTIGF	2188.875593	1325.862578	2029.541045	134372.5466
